# Crossmixed convolutional neural network for digital speech recognition

**DOI:** 10.1371/journal.pone.0302394

**Published:** 2024-04-26

**Authors:** Quoc Bao Diep, Hong Yen Phan, Thanh-Cong Truong

**Affiliations:** 1 Faculty of Mechanical - Electrical and Computer Engineering, Van Lang University, Ho Chi Minh City, Vietnam; 2 Faculty of Information Technology, University of Finance-Marketing, Ho Chi Minh City, Vietnam; Guangdong University of Petrochemical Technology, CHINA

## Abstract

Digital speech recognition is a challenging problem that requires the ability to learn complex signal characteristics such as frequency, pitch, intensity, timbre, and melody, which traditional methods often face issues in recognizing. This article introduces three solutions based on convolutional neural networks (CNN) to solve the problem: 1D-CNN is designed to learn directly from digital data; 2DS-CNN and 2DM-CNN have a more complex architecture, transferring raw waveform into transformed images using Fourier transform to learn essential features. Experimental results on four large data sets, containing 30,000 samples for each, show that the three proposed models achieve superior performance compared to well-known models such as GoogLeNet and AlexNet, with the best accuracy of 95.87%, 99.65%, and 99.76%, respectively. With 5-10% higher performance than other models, the proposed solution has demonstrated the ability to effectively learn features, improve recognition accuracy and speed, and open up the potential for broad applications in virtual assistants, medical recording, and voice commands.

## 1 Introduction

The recognition of acoustic features, mainly digital speech recognition, is essential in the present scientific domain. It has widespread application in diverse areas of academia and industry, such as bearing fault detection based on vibration analysis, earthquake early warning systems, voice commands, musical instrument classification, and numerous other practical implementations. However, current solutions still exhibit certain limitations, including slow algorithm processing speed, long training time, consuming many computer resources, and unsatisfactory accuracy. Therefore, these limitations restrict practical deployment, particularly on devices with low computational capabilities like mobile devices.

For instance, in machine failure detection, identifying engine-broken problems throughout vibration signals requires collecting a substantial amount of data and utilizing high-performance equipment to satisfy the training requirements of deep models with a significant number of parameters [1–4]. Consequently, this leads to a reduction in the solution’s capacity for flexible application. Within earthquake early warning systems, detecting subterranean vibration indications necessitates expeditious and accurate analysis and predictive capabilities [5, 6]. However, the acquisition and subsequent analysis of extensive sensor-derived data necessitate adherence to rigorous processing equipment prerequisites. As a result, the ability to promptly respond during an earthquake is diminished.

Analyzing audio signals in digital speech recognition applications necessitates proficiency in distinguishing and transcribing raw waveform into textual representations or different languages [7, 8]. The feature extraction of audio of diverse qualities and multiple languages undermines the efficacy and precision of speech recognition. Similarly, in music classification applications, identifying audio signals requires the ability to categorize and label distinct musical genres [9, 10]. The recognition of audio segments with differing durations and formats necessitates the utilization of high-powered devices to fulfill the classification requirements, thereby limiting the practical flexibility of the application.

Most traditional digital speech recognition models rely heavily on manual feature extraction, which requires expert knowledge and much effort. They usually have issues recognizing complex signal characteristics such as frequency, pitch, timbre, and melody. These challenges represent a significant barrier to building accurate, computationally efficient speech recognition systems.

Three different CNN architectures are introduced in this study to address the shortcomings above, namely: one-dimensional CNN (1D-CNN), two-dimensional straight CNN (2DS-CNN), and two-dimensional mixed CNN (2DM-CNN) for digital speech recognition. These models can learn directly from raw data without manual feature extraction. In particular, 2DS-CNN and 2DM-CNN use Fourier transform techniques to convert digital audio to such spectrogram or mel-spectrogram images, helping to effectively learn essential features. In addition, we take advantage of modern techniques such as dropout and regularization to optimize computing performance. The evaluation of these algorithms encompasses various criteria, such as accuracy, precision, recall, and f1-score.

The main contribution of the research is to propose three advanced CNN architectures, taking advantage of modern signal processing techniques to enhance digital speech recognition capabilities. Specifically, the contributions include: (1) Proposing three CNN architectures 1D-CNN, 2DS-CNN and 2DM-CNN for digital speech recognition; (2) Overcome limitations of manual feature extraction from raw waveform data; (3) Using Fourier transform to convert digital speech to spectrogram, mel-spectrogram images, improving feature learning, achieving better performance compared to other CNN models; Moreover, (4) Open up the potential for broad applications in virtual assistants, medical recording, and voice commands.

The remainder of the article is organized as follows: Section 2 reviews related research and points out the strengths and weaknesses of these methods; Section 3 presents solutions, including proposed convolutional neural networks that reduce the number of parameters and increase the performance; Section 4 describes the experimental setup conducted to evaluate the effectiveness and performance of the proposed solution; Section 5 presents the results and discussion; Section 6 concludes the work.

## 2 Related work

Several techniques have been proposed in the literature to recognize acoustic features. G. Tang et al. used the improved AecNet model to classify sound events with two different audio preprocessing methods, Scalemax and Mean 0 Std 1. They achieved a performance of 84.9% on ESC-10, 68.6% on ESC-50, and 86.5% on DCASE, respectively [11]. These results show that the model can operate well and does not increase computational cost with small-sized datasets. However, it may not be effective when classifying larger datasets. The proposed method should clearly explain the mechanism and reason for using 1*x*1 convolutions and concatenating feature layers.

J. Naranjo-Alcaza et al. conducted research on sound classification [12]. The author performed fine-tuning changes to six different types of residual blocks and performed on two datasets, UrbanSound8k and ESC-10, with two different audio preprocessing methods, and compared their performance. The effectiveness achieved on the RB4 residual block has the highest performance on both datasets, 68% for the UrbanSound8k dataset and 79% for the ESC-10 dataset. It showed that the proposed model can operate well on small-sized datasets. However, choosing the appropriate residual block design may depend heavily on the input data, affecting the model’s performance.

Q. Zhu and X. Zu focused on analyzing and comparing the performance of many different implementations of the residual block [13]. The main goal is to explore the suitability of different residual blocks designed for raw image classification. The model is implemented on 4 datasets, and the highest performance in the test is 77.82% on CIFAR100, 94.25% on FaceScrub, 82.06% on ImageNet(100), and 77.56% on miniImageNet, respectively. The proposed model reduces the number of computational parameters, improves the recognition rate, and increases the accuracy and stability of the model. However, the article has yet to compare with other image classification methods, has not tested the effectiveness of POD Loss on other datasets and network architectures, and has not clearly explained the mechanism and reason for using POD Loss in raw image classification.

The authors of the study [14] examined how well two machine learning models performed in identifying emotions from speech: random forest (RF) with RF-based feature selection and one-dimensional convolutional neural network (conv1D). By adding more audio files, the authors’ small dataset for speech-based emotion recognition is expanded. They compared the two models’ performance after extracting a variety of acoustic properties. According to the findings, conv1D obtains a lower average accuracy (69%) than RF with feature selection. It also has a lower precision (72%) and a higher recall (84%) for calm emotions than fear.

Tripathi and Mishra performed environmental sound classification to train a model to perform classification by sorting the shuffled parts of the audio spectrum to improve performance [15]. The performance of environmental sound classification achieved the highest classification result of 91.67% on ESC-10 and 75.09% on DCASE when using the model trained on the assumed task. However, determining an assumed task related to the target sound classification task is difficult. If the assumed task is unrelated to the target task, the model may not learn valuable features from the data.

A neural population-based convolutional neural network (CNN) model is presented in the paper [16] to mimic how the auditory cortex in the brain encodes genuine sounds. While squirrels were exposed to ambient noises, the activity of hundreds of individual neurons in their auditory cortex was observed by the scientists. They simultaneously predicted each of these neurons’ activity from the input audio spectrum using CNN. With a median predicted correlation coefficient of 0.67, our population CNN model explains 47% of the variance of the experimental data, showing it to perform much better than other models and classic linear-nonlinear models. More crucially, the trained CNN model demonstrates that it captures the common representation space for sound in the auditory cortex by being easily generalized to simulate new neurons not included in the original dataset.

T. Zhang et al. used mel-spectrogram decomposition combined with the CNN model to classify environmental sounds to improve classification performance [17]. The above method provides a new approach to sound scene classification and can take advantage of many unlabeled data. The classification performance achieved the highest result compared to 5 different models, 73.17% on DCASE. The paper has yet to compare with other sound feature extraction methods, such as mfcc, wavelet transform, or different CNN network architectures, such as ResNet, DenseNet, and Transformer. It has not surveyed the impact of model parameters and hyperparameters on classification performance.

In publication [18], Julia Berezutskaya et al. studied how the human brain processes sound in natural environments, for example, when watching movies. The authors collected EEG data from six patients with electrodes implanted during surgery while watching a 78-minute documentary. Next, they trained a deep artificial neural network (ANN) using the original audio tape of the movie to predict brain activity in response to the sound. This ANN model achieved high prediction accuracy, with Spearman correlation coefficients up to 0.5 in the lateral cingulate cortex. The model maintained good performance when applied to new data from 29 other patients watching a different movie, especially with dialogue. The sound features learned by the model reflect the acoustic properties specific to each type of sound, such as speech and music. They are consistent with the spatial and temporal distribution of neural activity.

Inik focused on optimizing hyperparameters using Particle Swarm Optimization (PSO) for CNNs to improve environmental sound classification performance [19]. Experiments were conducted on the ESC-10, ESC-50, and Urbansound8k datasets, achieving accuracies of 91.17%, 88.5%, and 74.85%, respectively. There is a difference in algorithm performance between data augmentation and non-augmentation. However, the article does not compare with other hyperparameter optimization methods or investigate the impact of PSO parameters and hyperparameters on optimization performance.

Jilt Sebastian et al. proposed in [20] a new signal-to-signal (S2S) neural network to estimate spike signals from calcium imaging data. This method takes the calcium fluorescence signal as input and learns to estimate the spike signal end-to-end. Experiments on the spikefinder challenge dataset show that the S2S method outperforms other state-of-the-art methods, achieving a Pearson correlation coefficient of 0.6404 on the test set, providing a 46% improvement over the best method in the competition and 2,832 times better than the baseline model. It also improves the rank correlation coefficient by 56% compared to the best baseline model, reaching 0.5208, and has an area under the ROC curve (AUC) equivalent to 0.847. The article also analyzes the generalization ability, reliability, training target dependence, and interpretability of the S2S method.

S. Abdoli et al. proposed using 1D-CNN to classify environmental sounds [21]. The network input is learned directly from raw audio signals, resulting in a classification performance on the UrbanSound8k dataset of 89% with a more compact architecture and fewer parameters than the 2D-CNN representation. It can exploit the temporal structure of the audio signal and learn filters suitable for the classification task. However, the feasibility and stability of the proposed method could not be evident when only tested on a single dataset.

Quamhan et al. performed a classification of audio devices and recording environments based on spectrogram feature extraction [22]. The article uniquely combined CNN and LSTM (CRNN) to thoroughly exploit the audio signal’s valuable spatial and temporal features. The result of environmental sound classification achieved the highest classification results, 98% and 98.57% for device classification on the KSU-DB dataset based on the influence of gender, phoneme type, environmental noise, and device quality on classification quality. However, the article only uses spectrogram feature extraction, performs a comparison with the traditional CNN model, and has yet to test the model on larger datasets.

I. Wieser et al. introduced two new methods to understand the audio representation of emotional expressions through neural networks [23]. Both methods allow for deep analysis and a better understanding of essential representations in emotional expression through speech. However, it has yet to explain more about what the neural network learns and how it does with the learned representations related to emotional characteristics in speech.

Greta Tuckute et al. presented research on the ability of deep neural network (DNN) models trained for audio processing tasks to predict human brain responses to natural sound [24]. The authors evaluated the correlation between the stages of the DNN model and brain regions involved in sound processing by comparing the model’s output with brain fMRI data. The results show that most DNN models predict brain responses better than the standard temporal-spectral filtering model and exhibit systematic correlations between model stages and brain regions. However, some of the most advanced models predict worse. Models trained to recognize speech in noisy environments showed better brain prediction results than models trained in quiet environments. The training task also influences the prediction quality for each brain response characteristic, with the best prediction results obtained from models trained on multiple tasks.

L. Gao et al. researched sound classification based on various feature representations [25]. Using three feature representations such as logMel, CQT, and mfcc, as inputs for neural networks and applying the knowledge transfer method to compress knowledge from multiple neural networks into a single neural network reduces the computational cost of the model. However, the article needs to indicate why using multiple feature representations can improve classification performance and survey the impact of parameters such as the number of representations, the number of neural networks, and the value of hyperparameters in the soft probability calculation equation.

A novel staircase network model is presented by Zhenqing Li et al. [26] to enhance speech interpretation and quality in noisy conditions. The model’s distinct architecture prevents gradient fading across layers and captures long-term correlations in voice data. To concentrate on important spectral regions, avoidance linkages and concentrating mechanisms are provided. As the training aim, the model is trained to estimate an ideal scaling mask (IRM). Based on experimental evaluations using TIMIT, LibriSpeech, and VoiceBank datasets, the proposed model improves STOI by 16.21%, 16.41%, and 18.33% compared to noisy speech; PESQ improved by 31.1%, 32.9%, and 32%. The suggested model performs better in conditions with known and unknown noise than previous DNN networks. With the Kaldi engine for automatic speech recognition, the model achieved an average word error rate of 15.13% in noisy environments.

Khurana et al. focused on sound classification based on Mel-frequency spectrogram representation [27]. The TiCNN network proposed by the author classifies emotions into 8 different types and achieved a performance of 93.27% on the RAVDESS dataset. However, it should explain more about what the network has learned from the emotional features of speech.

F. Demir et al. focused on sound classification based on the representation of Mel-frequency spectrograms [28]. The article used 3 different neural network models, AlexNet, VGG16, VGG19, and the SVM model, to classify heart sounds and achieved high performance on 2 datasets, FSDKaggle2018 and TUT Urban Acoustic Scenes 2018. However, it does not explain the impact of parameters such as window size, overlap, and the number of FFTs in creating spectrogram images. It needs to explain why transfer learning can be beneficial.

Although the mentioned publications have achieved high accuracy in acoustic feature recognition, the neural network architectures still need to be simplified, increasing calculation time and slowing down the system. In this research, the author proposes a more effective solution to address those limitations and improve the algorithm’s accuracy and stability.

## 3 Methodlogy

### 3.1 Visual representation of digital speech

Digital speech can be visualized as a figure representing a signal’s frequency spectrum. It contains the frequency components of sound and their intensity. In a frequency spectrum that appears as an RGB image, the horizontal axis typically describes frequencies from low to high, and the vertical axis illustrates the sound intensity corresponding to each contributed frequency. Intensity is often expressed using color or brightness.

**Fig 1:**
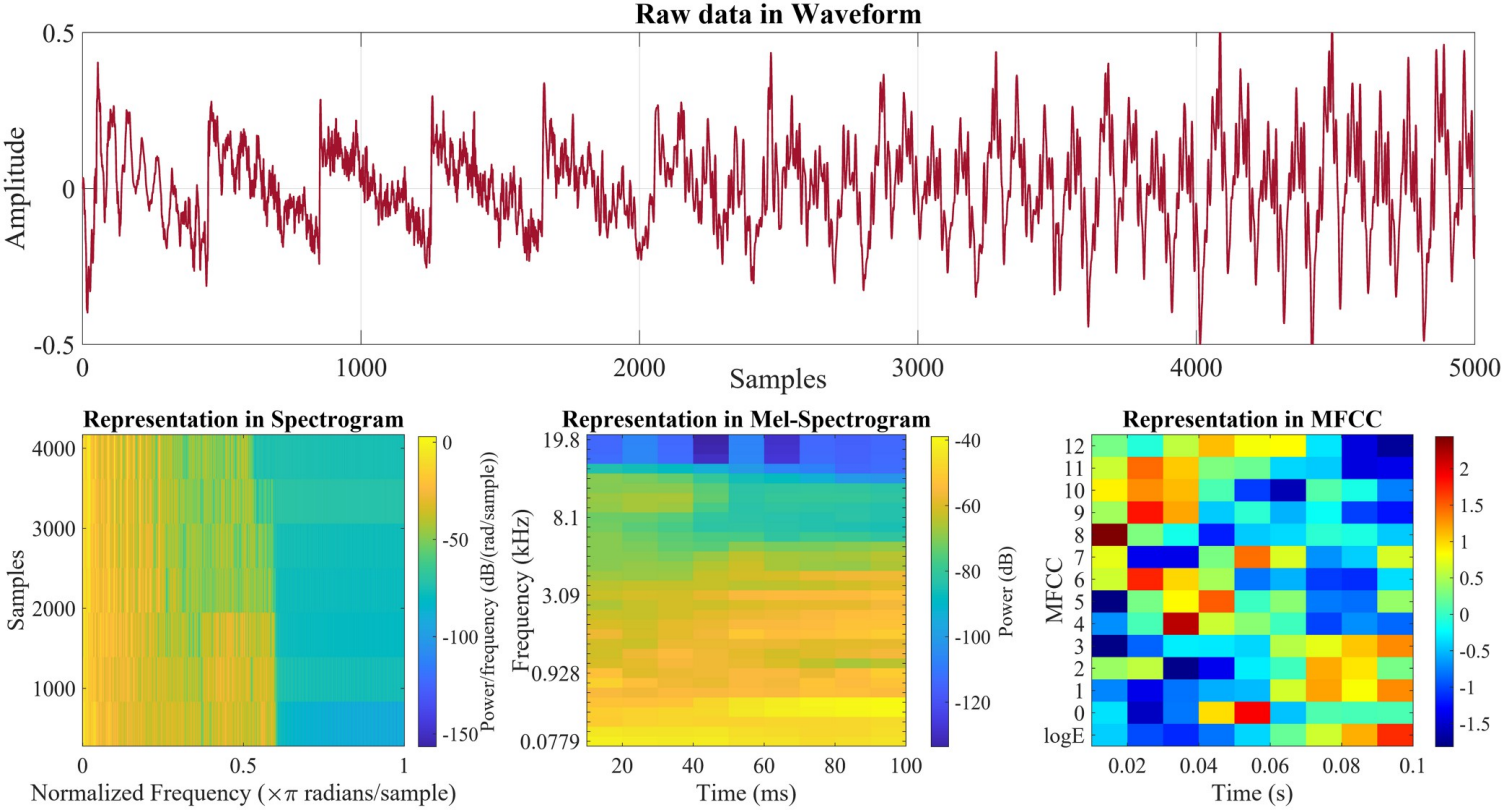
Raw data in waveform and representation in spectrogram, mel-spectrogram, and mfcc form.

The frequency spectrum reflects critical sound components and allows the analysis of characteristics such as scale, wave shape, resonance, and other phenomena. It is an essential mechanism in the study and analysis of sound. In this research, we propose to define digital speech through three visualization types: spectrogram, mel-spectrogram, and mfcc, as shown in Fig 1 and described in the next parts.

#### 3.1.1 Representation in spectrogram form

To represent digital speech as a spectrogram image, it is necessary to use Fourier transform to transform an audio signal from the time domain to the frequency domain. A typical transformation used for this purpose is the short-time Fourier transform, as described in Eq 1, more details in [29, 30]. The result is a spectrogram image where the horizontal axis represents time, the vertical axis represents frequency, and color or brightness represents sound intensity.

$$\begin{array}{c} \text{STFT}\left\{x\left(n\right)\right\}\left(m,\omega \right)\equiv X\left(m,\omega \right)=\sum_{n=-\infty }^{+\infty }x\left(n\right)w\left(n-m\right)e^{-i\omega n} \end{array}$$ (1)

where:

- **STFT**: Short-time Fourier transform,
- *X*_*m*_(*f*): Discrete-time **STFT**,
- *x*(*n*): the signal,
- *w*(*n* − *m*): the window function,
- *ω*: the frequency,
- *n*: discrete time.

#### 3.1.2 Representation in mel-spectrogram form

The mel-spectrogram uses a frequency scale called the mel scale to replace the linear frequency scale in conventional spectrograms, using the Eq 2 from O’Shaughnessy [31] to convert *f* hertz into *m* mels. The mel scale defines a nonlinear transformation, creating a frequency representation close to how humans hear.

$$\begin{array}{c} m=2595\;{\text{log}}_{10}\;\left(1+\frac{f}{700}\right) \end{array}$$ (2)

where:

- *m*: the mel value,
- *f*: the frequency.

#### 3.1.3 Representation in mfcc form

After applying the Fourier transform to the signal and replacing the linear frequency scale with the mel scale, we apply logarithm to each mel frequency of powers and then apply the Discrete Cosine Transform (DCT-II) of the list of mel log powers according to Eq 3, more details at [30].

$$\begin{array}{c} X\left(k\right)=\sum_{n=0}^{N-1}x\left(n\right)\;\text{cos}\;[\frac{\pi }{N}(n+\frac{1}{2})k],\;\text{for}\;k=0,...,N-1 \end{array}$$ (3)

### 3.2 Proposed convolutional neural networks

The proposed architecture aims to deal with digital speech recognition on both input types of digital speech (waveform) and transformed images (spectrogram, mel-spectrogram, and mfcc) in order to satisfy several expected requirements such as accuracy, run time, and robustness.

#### 3.2.1 One-dimensional convolutional neural network

One-dimensional convolutional neural network (1D-CNN) is a particular type of convolutional neural network designed to process one-dimensional data such as speech or time series data. The value of 1D-CNN is that it can learn directly from raw data, that is, original data that has not been processed or extracted features, and it can handle audio signals of any length. In the digital speech recognition problem, a 1D-CNN is used to automatically extract features from voices. The input to the 1D-CNN is a signal, and the output is a feature vector representing important information about the voice. Once trained on a given labeled speech dataset, the network can learn to distinguish between different voices or speakers.

**Fig 2:**
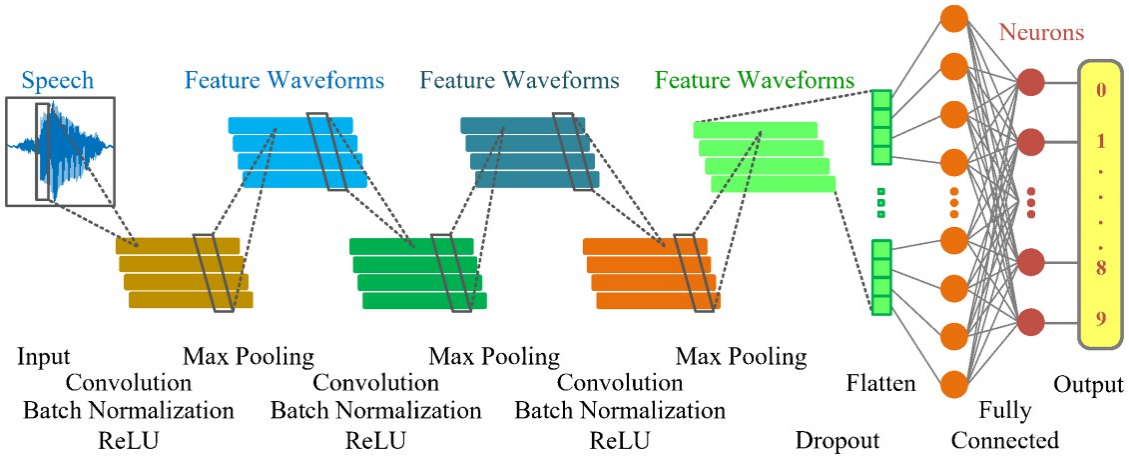
The architecture of the proposed 1D-CNN model.

The proposed 1D-CNN model contains three Convolution layers, in which the number of kernels (filter size) gradually increases from 10, 20, to 30 to extract features from input voice data. After the Convolution layers, the Batch Normalization, ReLU, Max Pooling, and Dropout layers are arranged in a specific order. Finally, two Fully Connected layers are used to learn the engineering features. To make classification decisions, the model uses Softmax and cross-entropy functions. The architecture of the proposed network is depicted in Fig 2, and the detailed parameters are given in Table 1.

**Table 1:** Layers and parameters of the proposed 1D-CNN model.

No.	Layer name, description	Channels	Learnale properies	Learnables
1	Sequence Input, with 1 dimention	1(c) x 1(B) x 1(T)	-	-
2	1-D Convolution, 10x100 with Stride 30, padding ‘same’	10(c) x 1(B) x 1(T)	Weights 100 x 1 x 10, Bias 1 x 10	1,010
3	Batch normalization	10(c) x 1(B) x 1(T)	Offset 10 x 1, Scale 10 x 1	20
4	ReLU	10(c) x 1(B) x 1(T)	-	-
5	1-D Max Pooling, with pool size 20, stride 20, padding ‘same’	10(c) x 1(B) x 1(T)	-	-
6	1-D Convolution, 20x100 with Stride 10, padding ‘same’	20(c) x 1(B) x 1(T)	Weights 100 x 10 x 20, Bias 1 x 20	20,020
7	Batch normalization	20(c) x 1(B) x 1(T)	Offset 20 x 1, Scale 20 x 1	40
8	ReLU	20(c) x 1(B) x 1(T)	-	-
9	1-D Max Pooling, with pool size 20, stride 20, padding ‘same’	20(c) x 1(B) x 1(T)	-	-
10	1-D Convolution, 30x100 with Stride 30, padding ‘same’	30(c) x 1(B) x 1(T)	Weights 100 x 20 x 30, Bias 1 x 30	60,030
11	Batch normalization	30(c) x 1(B) x 1(T)	Offset 30 x 1, Scale 30 x 1	60
12	ReLU	30(c) x 1(B) x 1(T)	-	-
13	1-D Max Pooling, with pool size 20, stride 20, padding ‘same’	30(c) x 1(B) x 1(T)	-	-
14	1-D global Max Pooling	30(c) x 1(B)	-	-
15	Dropout, 20% dropout	30(c) x 1(B)	-	-
16	Fully Connected, 100 fully connected layer	100(c) x 1(B)	Weights 100 x 30, Bias 100 x 1	3,100
17	Fully Connected, 10 fully connected layer	10(c) x 1(B)	Weights 10 x 100, Bias 10 x 1	1,010
18	Softmax	10(c) x 1(B)	-	-
19	Classification Output, crossentropyex	10(c) x 1(B)	-	-
-	Total learnables	-	-	85,290

#### 3.2.2 Two-dimensional straight convolutional neural network

Two-dimensional straight convolutional neural network (2DS-CNN) is an improved version of the traditional network, designed to process data such as spectrogram images. Convolutional layers work by sliding over vertical and horizontal areas of images to extract engineering features. Each following layer in this chain learns from the previous layer’s output, constructing a line through the network and allowing the network to learn increasingly complex features. The proposed network architecture is designed with two Convolution layers with kernels of 25 and 40. After each Convolution layer is Batch Normalization, ReLU, Max Pooling, and Dropout layers are arranged to form a straight line capable of extracting features from input images, including width, height, and color space, allowing the model to understand and gain more clarity about the data and improve classification performance. Finally, there are two Fully Connected layers to classify images. The model uses the Softmax and the cross-entropy function to make classification.

**Fig 3:**
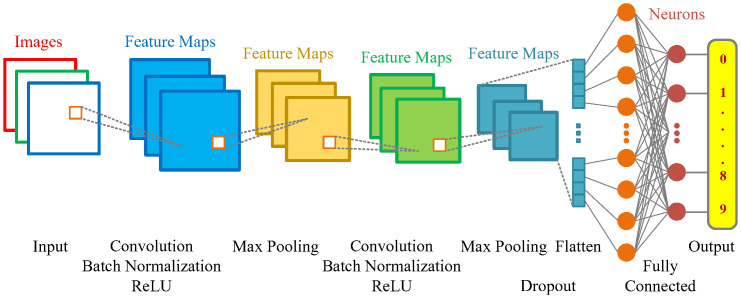
The architecture of the 2DS-CNN model.

The 2DS-CNN network architecture used in this study is presented in Fig 3, and the parameters are shown in the Table 2.

**Table 2:** Layers and parameters of the 2DS-CNN model.

No.	Layer name, description	Channels	Learnale properies	Learnables
1	Image Input, 224 x 224 x 3 images, ‘rescale-zero-one’ normalization	224(s) x 224(s) x 3(c)	-	-
2	2-D Convolution, 25 5x5 Convolutions with Stride [3 3], padding ‘same’	75(s) x 75(s) x 25(c)	Weights 5 x 5 x 3 x 25, Bias 1 x 1 x 25	1,900
3	Batch normalization	75(s) x 75(s) x 25(c)	Offset 1 x 1 x 25, Scale 1 x 1 x 25	50
4	ReLU	75(s) x 75(s) x 25(c)	-	-
5	2-D Max Pooling, 2 x 2 max pooling with stride [2 2], padding [0 0 0 0]	37(s) x 37(s) x 25(c)	-	-
6	2-D Convolution, 40 3x3 Convolutions with Stride [2 2], padding [0 0 0 0]	18(s) x 18(s) x 40(c)	Weights 3 x 3 x 25 x 40, Bias 1 x 1 x 40	9,040
7	Batch normalization	18(s) x 18(s) x 40(c)	Offset 1 x 1 x 40, Scale 1 x 1 x 40	80
8	ReLU	18(s) x 18(s) x 40(c)	-	-
9	2-D Max Pooling, 2 x 2 max pooling with stride [2 2], padding [0 0 0 0]	9(s) x 9(s) x 40(c)	-	-
10	Dropout, 20% dropout	9(s) x 9(s) x 40(c)	-	-
11	Fully Connected, 100 fully connected layer	1(s) x 1(s) x 100(c)	Weights 100 x 3240, Bias 100 x 1	324,100
12	Fully Connected, 10 fully connected layer	1(s) x 1(s) x 10(c)	Weights 10 x 100, Bias 10 x 1	1,010
13	Softmax	1(s) x 1(s) x 10(c)	-	-
14	Classification Output, crossentropyex	1(s) x 1(s) x 10(c)	-	-
-	Total learnables	-	-	336,180

#### 3.2.3 Crossmixed convolutional neural network

A crossmixed convolutional neural network (2DM-CNN) is an efficient architecture composed of two types of layers: serial and parallel. Serial layers allow the network to learn increasingly complex features as it goes deeper into the network, and parallel layers allow it to learn features at the same level but with different filters, creating diversity in learning. Such architecture allows the network to learn both low-level and high-level features.

The 2DM-CNN architecture is divided into 2 blocks. The first block contains three parallel Convolution layers with kernel sizes of 3×3, 5×5, and 7×7, respectively. It extracts features at different spatial scales from the input image, increasing the model’s ability to recognize features. Each Convolution layer is followed by a Max Pooling layer, which reduces the output size of each previous layer and increases calculation speed. The outputs of all three Max Pooling layers are then combined by stacking them via a Depth Concatenation layer. The second block consists of output results from the first Depth Concatenation layer as input for the following three parallel Convolution layers with kernel sizes of 7×7, 5×5, and 3×3, respectively, to carry out the task of feature extraction at many different spatial scales. A ReLU layer and a Max Pooling layer follow each Convolution layer. Next, stack the results at the Max Pooling output through the second Depth Concatenation layer and connect with two Fully Connected layers to classify the data. Finally, the model applies the Softmax function to generate probability values for each prediction class and uses the cross-entropy function to evaluate the confidence of the classification.

**Fig 4:**
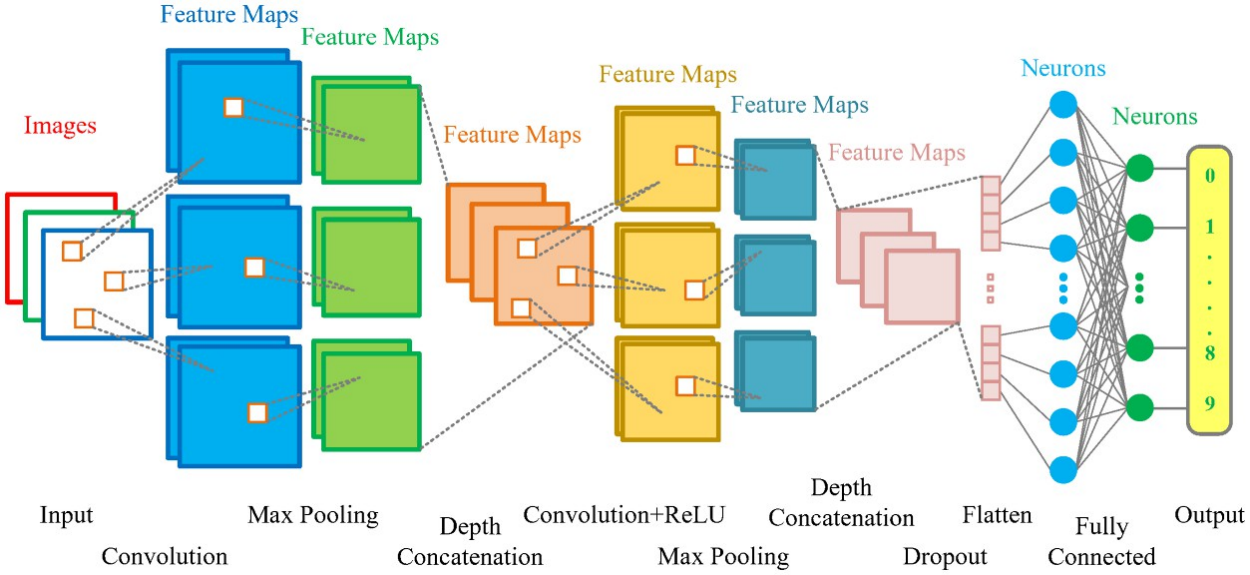
The architecture of the 2DM-CNN model.

The 2DM-CNN network architecture used in this study is presented in Fig 4, and parameters are shown in Table 3.

**Table 3:** Layers and parameters of the 2DM-CNN model.

No.	Layer name, description	Channels	Learnale properies	Learnables
1	Image Input, 224 x 224 x 3 images	224(s) x 224(s) x 3(c)	-	-
2	Batch normalization	224(s) x 224(s) x 3(c)	Offset 1 x 1 x 3, Scale 1 x 1 x 3	6
3	2-D Convolution, 16 3x3 Convolutions with Stride [2 2], padding [0 0 0 0]	111(s) x 111(s) x 15(c)	Weights 3 x 3 x 3 x 16, Bias 1 x 1 x 16	448
4	2-D Max Pooling, 7 x 7 max pooling with stride [7 7], padding [0 0 0 0]	15(s) x 15(s) x 16(c)	-	-
5	2-D Convolution, 16 5x5 Convolutions with Stride [3 3], padding [0 0 0 0]	74(s) x 74(s) x 16(c)	Weights 5 x 5 x 3 x 16, Bias 1 x 1 x 16	1,216
6	2-D Max Pooling, 5 x 5 max pooling with stride [5 5], padding [0 0 0 0]	15(s) x 15(s) x 16(c)	-	-
7	2-D Convolution, 16 7x7 Convolutions with Stride [5 5], padding [0 0 0 0]	45(s) x 45(s) x 16(c)	Weights 7 x 7 x 3 x 16, Bias 1 x 1 x 16	2,368
8	2-D Max Pooling, 3 x 3 max pooling with stride [3 3], padding [0 0 0 0]	15(s) x 15(s) x 16(c)	-	-
9	Depth Concatenation, Depth concatenation of 3 inputs	15(s) x 15(s) x 48(c)	-	-
10	2-D Convolution, 32 5x5 Convolutions with Stride [3 3], padding ‘same’	5(s) x 5(s) x 32(c)	Weights 5 x 5 x 48 x 32, Bias 1 x 1 x 32	38,432
11	ReLU	5(s) x 5(s) x 32(c)	-	-
12	2-D Max Pooling, 3 x 3 max pooling with stride [3 3], padding ‘same’	2(s) x 2(s) x 32(c)	-	-
13	2-D Convolution, 32 3x3 Convolutions with Stride [2 2], padding ‘same’	8(s) x 8(s) x 32(c)	Weights 3 x 3 x 48 x 32, Bias 1 x 1 x 32	13,856
14	ReLU	8(s) x 8(s) x 32(c)	-	-
15	2-D Max Pooling, 4 x 4 max pooling with stride [4 4], padding ‘same’	2(s) x 2(s) x 32(c)	-	-
16	2-D Convolution, 32 7x7 Convolutions with Stride [5 5], padding ‘same’	3(s) x 3(s) x 32(c)	Weights 7 x 7 x 48 x 32, Bias 1 x 1 x 32	75,296
17	ReLU	3(s) x 3(s) x 32(c)	-	-
18	2-D Max Pooling, 2 x 2 max pooling with stride [2 2], padding ‘same’	2(s) x 2(s) x 32(c)	-	-
19	Depth Concatenation, Depth concatenation of 3 inputs	2(s) x 2(s) x 96(c)	-	-
20	Dropout, 20% dropout	2(s) x 2(s) x 96(c)	-	-
21	Fully Connected, 50 fully connected layer	1(s) x 1(s) x 50(c)	Weights 50 x 384, Bias 100 x 1	19,250
22	Fully Connected, 10 fully connected layer	1(s) x 1(s) x 10(c)	Weights 10 x 50, Bias 10 x 1	510
23	Softmax	1(s) x 1(s) x 10(c)	-	-
24	Classification Output, crossentropyex	1(s) x 1(s) x 10(c)	-	-
-	Total learnables	-	-	151,382

## 4 Experimental setup

### 4.1 Dataset

To thoroughly evaluate the performance of the proposed models, four types of datasets were used. The first one is digital speech dataset, containing English spoken of ten digits from 0 to 9 [32]. The dataset includes 30,000 speech samples of 60 separate speakers of different ages, countries, genders, and recording environments. They pronounce each number 50 times, using a single-channel microphone with a sampling rate of 48kHz. The total duration of the dataset is approximately 9.5 hours.

**Fig 5:**
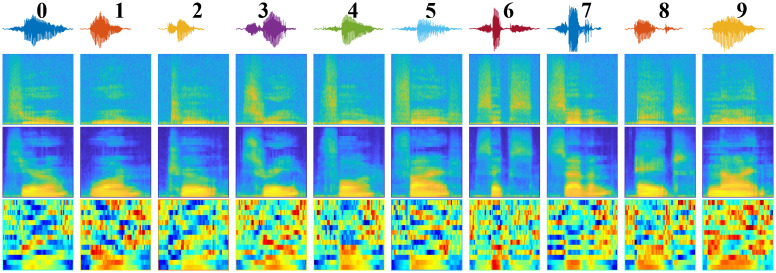
Speech, spectrogram, mel-spectrogram and mfcc images of used datasets.

The second type is three transformed image datasets: spectrogram, mel-spectrogram, and mfcc. Each set contains 30,000 images transformed from the digital speech dataset using the method presented in Subsection 3.1. There are a total of 10 classes, each labeled from 0 to 9, and contains 3,000 samples. Fig 5 provides the first sample of each dataset used.

Each dataset has been randomly split into three separate subsets: 70% for training, 10% for validation, and 20% for testing.

### 4.2 Models for comparison

To comprehensively evaluate the proposed models, five algorithms were selected for comparison in terms of performance, accuracy, and other critical metrics, including:

- Multi-layer pooling classifier convolutional neural network (MLPC-CNN) [3]: an algorithm designed to diagnose errors for multi-sensor vibration signals. It has been widely used in many applications related to vibration signals, from machine fault diagnosis to vibration signal analysis in different situations;
- Improved convolutional neural network for acoustic event classification (AecNet) [11]: designed to classify acoustic events and has been widely used in many applications related to sound, from speech recognition to environmental sound classification;
- End-to-End 1D convolutional neural network (End2End) [21]: an algorithm designed to classify environmental sounds and has been widely used in many applications related to sound, from speech recognition to environmental sound classification;
- GoogLeNet [33]: was designed to handle image classification tasks. GoogLeNet has been proven to be particularly effective in image classification, achieving top results in the Large Scale Image Recognition Contest ImageNet (ILSVRC) 2014;
- AlexNet [34]: was designed to handle image classification tasks. AlexNet achieved top results in the 2012 ILSVRC.

For GoogLeNet and AlexNet, the transfer learning technique was used to solve the digital speech recognition problem in this study. AlexNet received the input image with the size of 224 × 224 × 3 and was modified with 10 output layers, each corresponding to a digit. Similarly, GoogLeNet also receives the same input and output size as AlexNet.

### 4.3 Setup and metrics

Table 4 provides an overview of the hardware and software parameters employed in this study. The experiments were designed, performed, evaluated, and analyzed on MATLAB R2023a environment.

**Table 4:** Hardware specification values were used for training, testing, and analysis.

Items	Specification
Processor	CPU 13th Gen Intel(R) Core(TM) i7-13700KF Base speed: 3.40 GHz
RAM Memory	Memory 64.0 GB, Speed: 3600 MHz, Slots used: 4 of 4
OS	Edition: Windows 11 Pro, Version 23H2
System Model and Type	64-bit operating system, x64-based processor
GPU	GPU NVIDIA GeForce RTX 3060 Ti, DirectX version: 12 (FL 12.1)

All models are run iteratively 10 times on corresponding datasets (digital speech, spectrogram, mel-spectrogram, and mfcc) for all processes of training, testing, and evaluating obtained results. The results are calculated and compared based on the mean and standard deviation. The Wilcoxon rank sum test (WRT) was performed to ensure the comparison results were statistically significant at the 5% level [35, 36].

The parameter values of the proposed convolutional neural networks have been specified in Subsection 3.2.1, Subsection 3.2.2, and Subsection 3.2.3. The parameter values of the neural networks used for comparison were set similarly to those in the original published article and cited in Section 4.2.

All models were trained using the adaptive moment estimation optimizer, 10% validation, Initial Learning Rate of 0.0005, Max Epochs of 50, and Mini Batch Size of 500.

In order to rigorously evaluate the effectiveness of the proposed solutions, we investigate the models from two major aspects: the complexity aspect (**Space complexity, time complexity, and number of parameters**) and the performance aspect. Four main metrics are reported to evaluate and compare the performance of the model on the test dataset:

**Accuracy** denotes the proportion of accurate predictions relative to all predictions, which helps to understand the model’s overall performance. However, accuracy only sometimes reflects the model’s proper performance, especially when the data is imbalanced.
**Recall** denotes the proportion of accurate positive predictions relative to all actual positives; this index is high, meaning the model can detect many positive cases and miss a few critical cases.
**Precision** denotes the proportion of accurate positive predictions relative to all positive predictions; this index is high, meaning the model is less confused between classes and highly reliable when predicting a class.
**F1-Score** denotes a metric to assess the balance between two fundamental performance measures of recall and precision that helps to evaluate the model when both precision and recall need to be considered.

## 5 Results and discussion

### 5.1 Computational complexity

**Fig 6:**
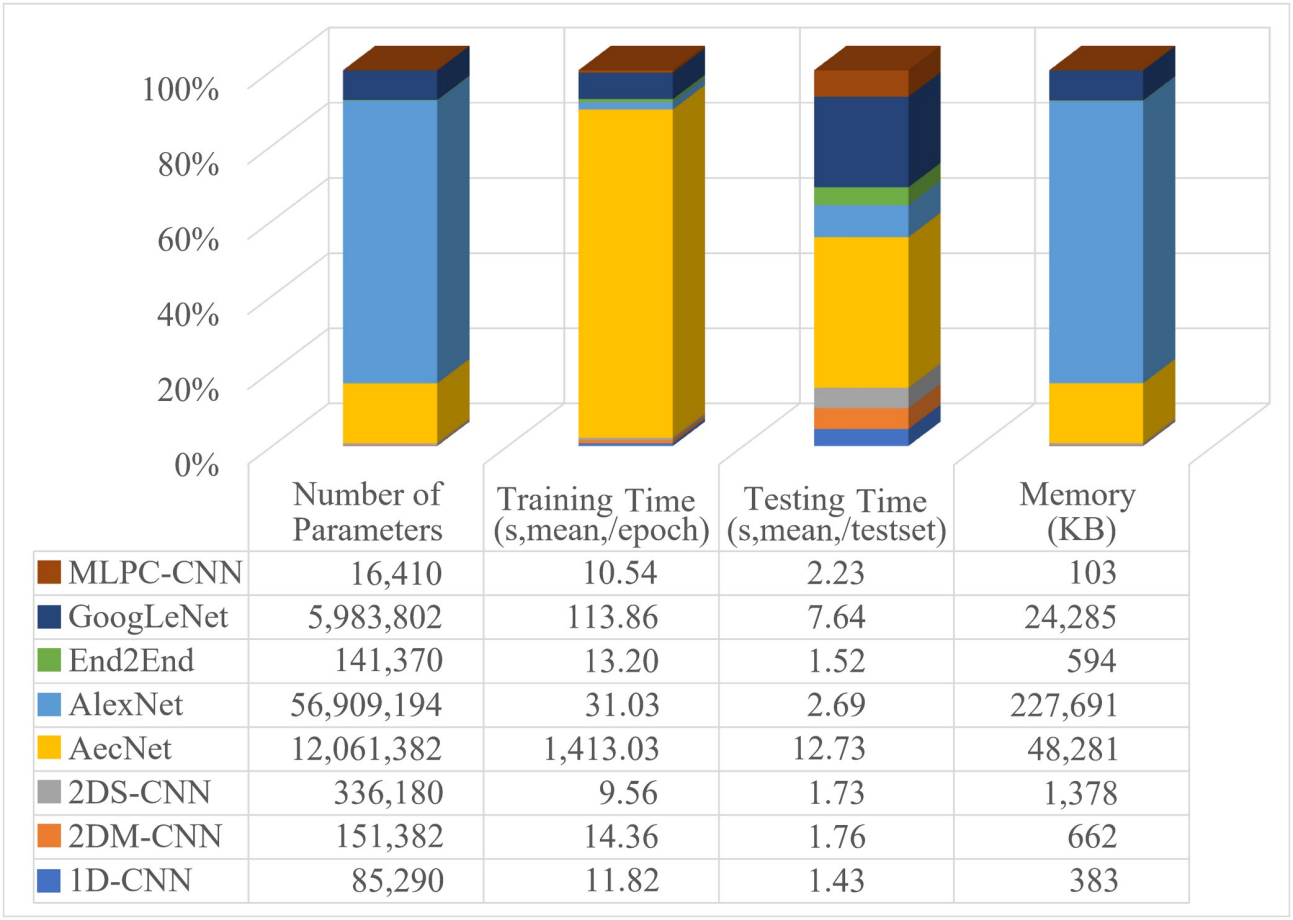
Comparison of complexity and time parameters of all models (lower is better).

Fig 6 compares the proposed models’ computational complexity to evaluate their practical feasibility. It reflects the detailed number of parameters, the amount of memory occupied (KB), and the execution time (s) on all models’ training/testing datasets. Including three main factors presented as follows:

**Space complexity**: reflects the amount of memory needed to contain model parameter storage space, input/output data, and intermediate memories during the calculation process. The 1D-CNN and 2DM-CNN models have lower space complexity due to using one- and two-dimensional convolution with bigger kernel sizes and strides. In contrast, 2DS-CNN uses smaller kernel sizes and strides and a bigger number of input channels, requiring more memory. Accordingly, the spatial complexity ranked in ascending order is: MLPC-CNN, 1D-CNN, End2End, 2DM-CNN, 2DS-CNN, GoogLeNet, AecNet, AlexNet.

**Time complexity**: measures the total time required to perform calculations during training and testing. It depends on the number of parameters, layers, size of each layer, number of iterations per epoch, etc. Among the proposed models, 2DS-CNN has the lowest time complexity during training, while 1D-CNN and 2DM-CNN are more time-consuming. On the other side, 1D-CNN performs fastest on the testing data. Meanwhile, AecNet consumes the most time in both training and testing.

Fig 6 also reports the time complexity details for all investigated models. It shows the training time on the training set arranged in ascending order as 2DS-CNN, MLPC-CNN, 1D-CNN, End2End, 2DM-CNN, AlexNet, GoogleNet, AecNet. As for the time complexity during testing on the testing set, this order varies and is arranged in ascending order as follows: 1D-CNN, End2End, 2DS-CNN, 2DM-CNN, MLPC-CNN, AlexNet, GoogLeNet, AecNet.

**Number of parameters**: reflects the complexity of the model; the larger the number, the higher the computational resources. This figure highlights the correlation between model performance and used resources. It shows that the GoogLeNet, AecNet, and AlexNet models have numerous parameters, over 5, 12, and 56 million parameters, respectively, leading to extensive calculation time, slow training speed, and taking up much memory. Meanwhile, our proposed networks have fewer parameters, ranging under 0.4 million, equivalent to 2.5% compared to GoogLeNet and 0.26% to AlexNet.

The 1D-CNN model uses one-dimensional convolutional layers with smaller learnable parameters than two-dimensional convolutional layers, so it has the near-fewest parameters (85,290), just behind the MLPC-CNN model with 16,410 parameters. The 1D-CNN model also has the fastest calculating time per whole test set of about 1.43s (mean value of 10 runs).

The MLPC-CNN model has the smallest number of parameters; on the other hand, it also has a high testing time of 2.23s. The 2DS-CNN and 2DM-CNN models have a higher number of parameters than 1D-CNN, End2End, and MLPC-CNN but are still much lower than three other models: AecNet, GoogLeNet, and AlexNet.

### 5.2 Performance comparison

Table 5 describes the performance comparison results of two 1D-CNN and End2End models performed on speech data. The 1D-CNN model performs better than the End2End model on all metrics, and the difference is statistically significant (*ρ*-value is less than 5%). That means the 1D-CNN model can learn and classify speech data better than the End2End model. Table 5 also shows that the standard deviation is slight, meaning that the difference between runs is negligible; the model performs stably, especially for the proposed 1D-CNN.

**Table 5:** Comparison results on digital speech dataset (mean±std).

Ranking	Models	Input type	Accuracy	Precision	Recall	F1-Score	*ρ*-value
1	1D-CNN	Speech	95.87 ± 0.48	95.87 ± 1.67	95.93 ± 2.40	95.88 ± 1.48	-
2	End2End	Speech	94.24 ± 0.23	94.24 ± 2.64	94.34 ± 3.65	94.26 ± 2.67	1.81e-04

Table 6 describes the comparison and ranking results of the surveyed models on the spectrogram image dataset. Three models achieve similar first-ranking results: 2DS-CNN, GoogLeNet, and 2DM-CNN. These models have accuracy, precision, recall, and f1-Score metric close to 99.20%. Other models have lower accuracy and statistically significant differences than the first-ranked model (*ρ*-value less than 0.05). Besides, Table 6 shows that the standard deviation of the proposed models is minimal (less than 1%), proving that the proposed models work more stably on the spectrogram dataset compared to the digital speech dataset.

**Table 6:** Comparison results on spectrogram dataset.

Ranking	Models	Input type	Accuracy	Precision	Recall	F1-Score	*ρ*-value
1	2DS-CNN	Spectrogram	99.20 ± 0.06	99.20 ± 0.49	99.20 ± 0.52	99.20 ± 0.37	-
1	GoogLeNet	Spectrogram	99.20 ± 0.17	99.20 ± 0.72	99.20 ± 0.80	99.20 ± 0.47	8.79e-01
1	2DM-CNN	Spectrogram	99.14 ± 0.07	99.14 ± 0.54	99.14 ± 0.73	99.14 ± 0.45	7.34e-02
2	AlexNet	Spectrogram	98.60 ± 0.06	98.60 ± 0.30	98.60 ± 0.36	98.60 ± 0.25	1.66e-04
3	AecNet	Spectrogram	97.47 ± 0.23	97.47 ± 1.61	97.52 ± 1.93	97.48 ± 1.22	1.75e-04
4	MLPC-CNN	Spectrogram	96.48 ± 0.01	96.48 ± 0.95	96.51 ± 1.46	96.49 ± 0.75	6.11e-05

Table 7 shows the comparison and ranking results of different models on the mel-spectrogram dataset. They are ranked in descending order of accuracy metric, and if equal, in descending order of f1-score. The proposed 2DM-CNN model achieves the highest performance, with an accuracy of 99.76%. MLPC-CNN is the network with the lowest performance, with an accuracy of 98.83%. Others, such as GoogLeNet, 2DS-CNN, AecNet, and AlexNet, have high performance, above 98%, and are lower than the 2DM-CNN model. The *ρ*-values show that the differences between models are statistically significant at the 5% level.

**Table 7:** Comparison results on the mel-spectrogram dataset.

Ranking	Models	Input type	Accuracy	Precision	Recall	F1-Score	*ρ*-value
1	2DM-CNN	Mel-spectrogram	99.76 ± 0.03	99.76 ± 0.29	99.76 ± 0.31	99.76 ± 0.19	-
2	GoogLeNet	Mel-spectrogram	99.66 ± 0.03	99.66 ± 0.22	99.66 ± 0.32	99.66 ± 0.16	2.20e-04
2	2DS-CNN	Mel-spectrogram	99.65 ± 0.05	99.65 ± 0.36	99.65 ± 0.34	99.65 ± 0.25	2.63e-04
3	AecNet	Mel-spectrogram	98.96 ± 0.13	98.96 ± 0.94	98.96 ± 0.93	98.96 ± 0.59	1.70e-04
4	AlexNet	Mel-spectrogram	98.94 ± 0.02	98.94 ± 0.10	98.94 ± 0.10	98.94 ± 0.07	1.62e-04
5	MLPC-CNN	Mel-spectrogram	98.83 ± 0.01	98.83 ± 0.42	98.84 ± 0.59	98.83 ± 0.40	5.84e-05

Table 8 shows the comparison and ranking results of all models on the mfcc dataset. The 2DM-CNN model achieved the highest performance, with an accuracy of 99.48%. The 2DS-CNN model ranked second, with 99.29% accuracy. The models applying transfer learning techniques like GoogLeNet and AlexNet ranked third and fourth, with an accuracy of 98.86% and 97.89%, respectively. The MLPC-CNN ranked last, with the lowest accuracy of 97.53%. The *ρ*-values show that the differences between the networks are statistically significant.

**Table 8:** Comparison results on the mfcc dataset.

Ranking	Models	Input type	Accuracy	Precision	Recall	F1-Score	*ρ*-value
1	2DM-CNN	MFCC	99.48 ± 0.07	99.48 ± 0.38	99.48 ± 0.38	99.48 ± 0.26	-
2	2DS-CNN	MFCC	99.29 ± 0.06	99.29 ± 0.47	99.29 ± 0.44	99.29 ± 0.36	1.75e-04
3	GoogLeNet	MFCC	98.86 ± 0.15	98.86 ± 0.93	98.88 ± 1.01	98.86 ± 0.47	1.76e-04
4	AlexNet	MFCC	98.81 ± 0.03	98.81 ± 0.16	98.81 ± 0.15	98.81 ± 0.10	1.72e-04
5	AecNet	MFCC	97.89 ± 0.17	97.89 ± 1.02	97.90 ± 1.05	97.89 ± 0.70	1.76e-04
6	MLPC-CNN	MFCC	97.53 ± 0.01	97.53 ± 1.06	97.53 ± 1.09	97.53 ± 1.00	6.15e-05

**Fig 7:**
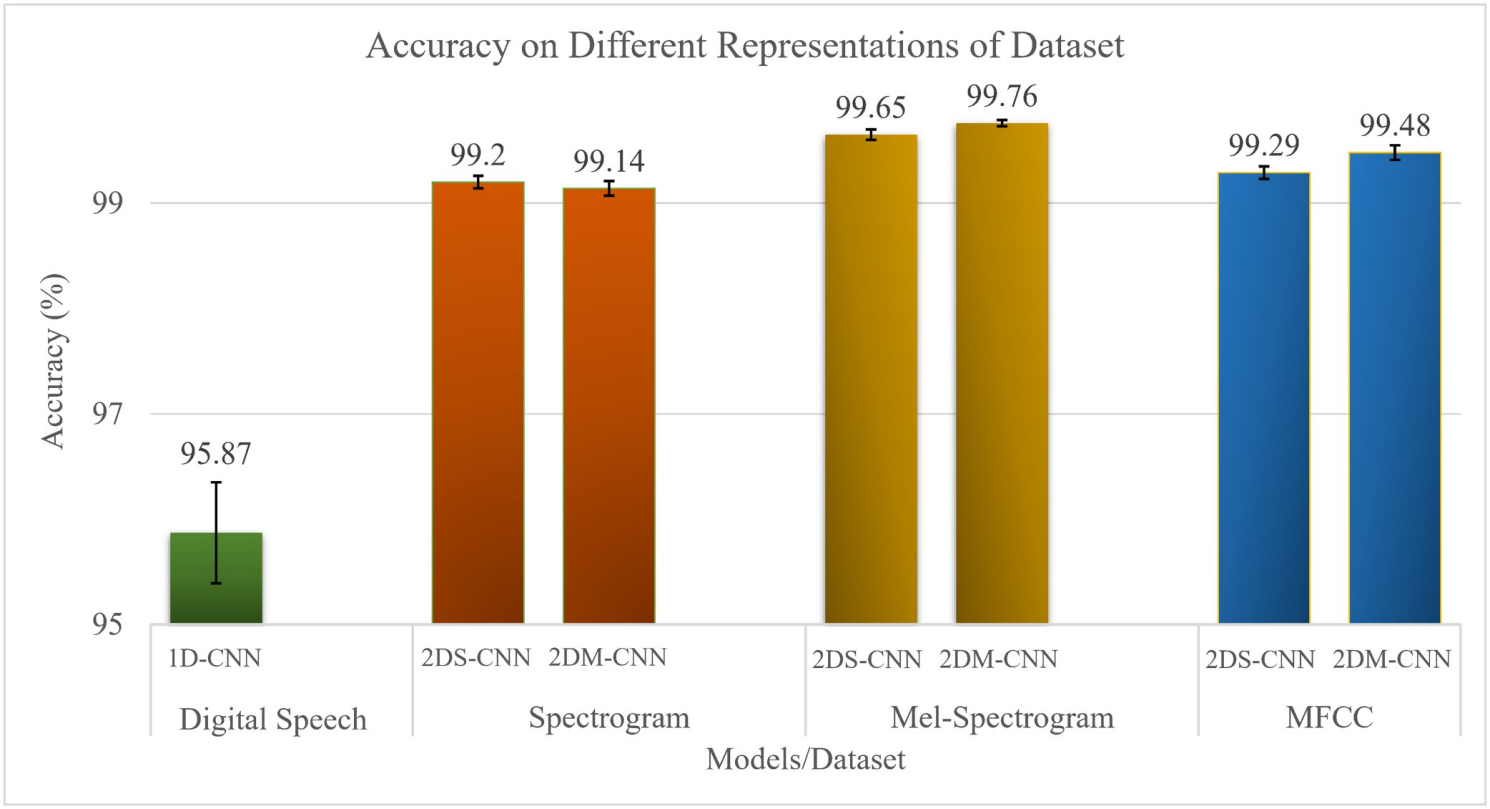
Results of the proposed algorithms (higher is better).

Fig 7 shows the classification performance (accuracy with mean and standard deviation) of the three proposed models, 1D-CNN, 2DS-CNN, and 2DM-CNN, on four types of the dataset, including digital speech for 1D-CNN, spectrogram, mel-spectrogram, and mfcc for the remaining two models. The classification results of the 2DM-CNN model on the mel-spectrogram dataset reach the highest performance of 99.76%. Next, the performance of the 2DS-CNN model on mel-spectrogram is 99.65%. Finally, the lowest classification performance is 1D-CNN, with an accuracy of 95.87%.

The results in Fig 7 show that digital speech recognition on the transformed image dataset will give higher accuracy and more stability than on the original digital speech dataset. The mel-spectrogram has the highest accuracy among the image datasets compared to the spectrogram and mfcc.

The difference in input type is also a critical factor that determines the classification performance of the models. For the raw waveform (speech data), both 1D-CNN and End2End models can learn the amplitude and duration of the digital speech, not other components, either frequency or intensity of the speech, and other essential features such as pitch, timbre, and melody. For the remaining input types, using more complex feature extractions such as spectrogram, mel-spectrogram, and mfcc yields better results than the 1D-CNN model, as shown in Fig 7. Spectrograms represent multiple sound components over time and reflect fundamental characteristics using different colors. Therefore, a spectrogram can extract more complex features of digital speech. However, it is vastly affected by noise or environmental background.

The mel-spectrogram represents digital speech in the frequency domain but uses the mel frequency scale instead of the regular frequency scale. Mel-spectrogram can extract features close to how humans hear and can overcome some limitations of the spectrogram, such as being unable to distinguish speech with the same pitch but a different timbre. So, the classification performance on the mel-spectrogram dataset is better.

**Fig 8:**
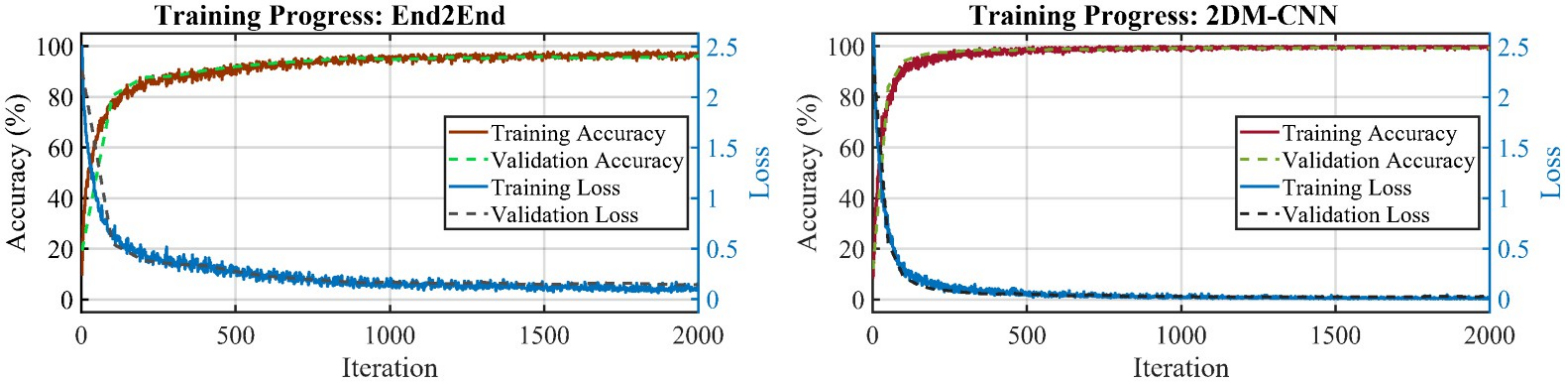
The training process of the End2End and 2DM-CNN models in the first run.

Fig 8 compares the training progress of End2End and 2DM-CNN models in the first run. The accuracy and loss curves of the 2DM-CNN model quickly approach 100% and 0, respectively, in about 500 iterations. Meanwhile, the convergence speed of the End2End model is slower: the final accuracy is lower (93.6% versus 99.79%), and the loss is higher than the 2DM-CNN model at iteration 2000.

**Fig 9:**
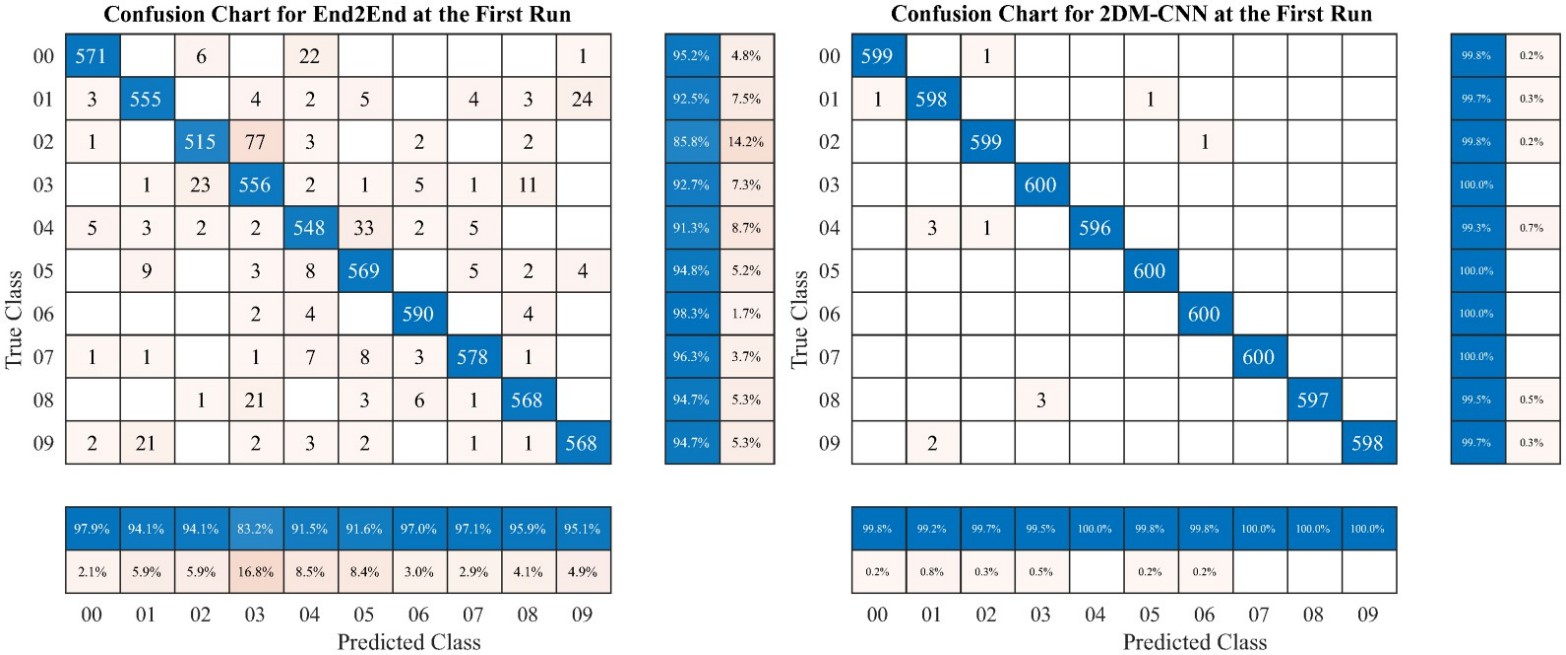
The confusion matrix of End2End and 2DM-CNN models in the first run.

Fig 9 depicts the confusion matrices, showing the classification results of End2End and 2DM-CNN. The last columns and rows of the matrix represent precision and recall values, respectively. It shows that 2DM-CNN gives the best classification results in the first run, whereas End2End performs much lower than 2DM-CNN.

**Fig 10:**
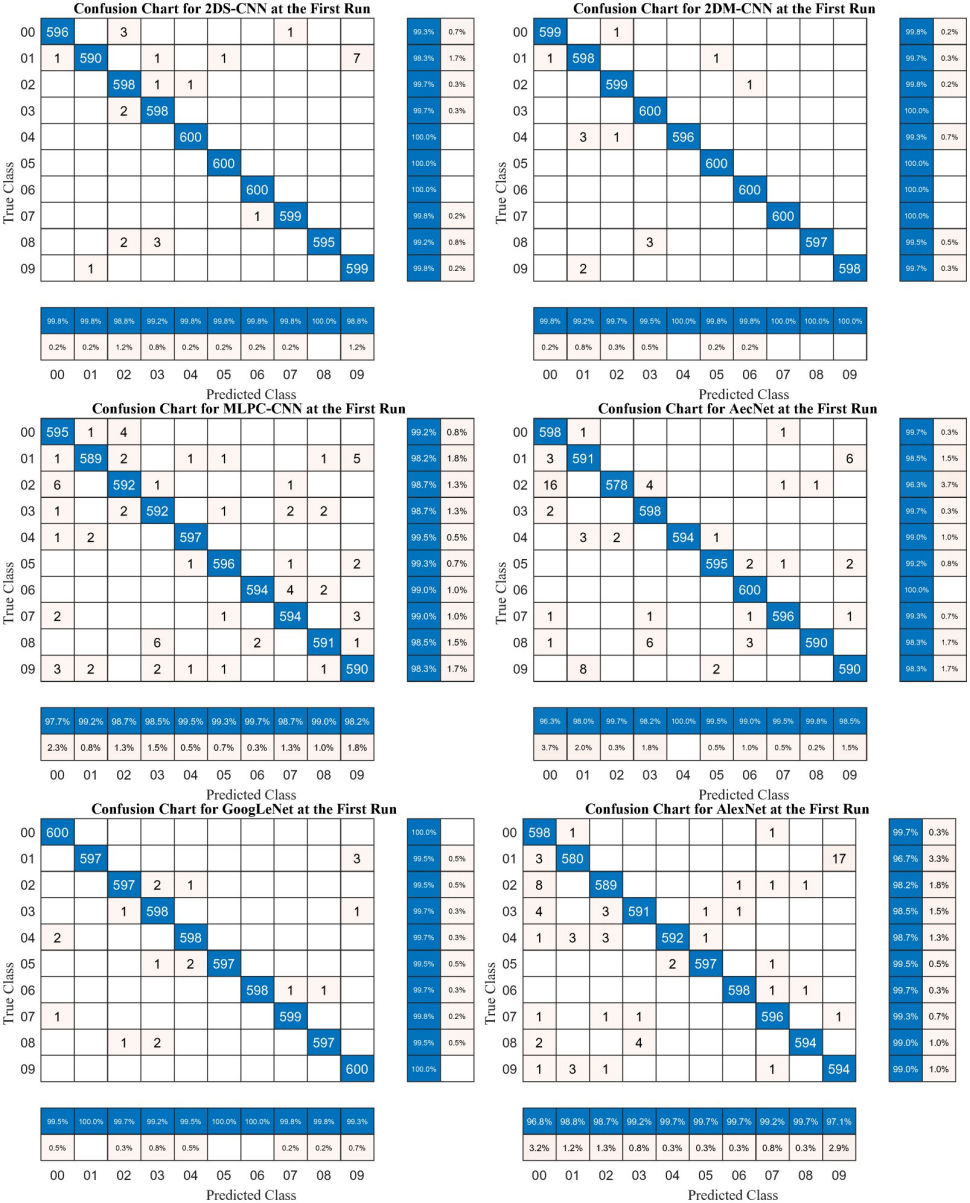
Confusion matrices at the first run of the proposed neural networks used on the mel-spectrogram dataset.

Fig 10 shows the confusion matrices and the classification results on the mel-spectrogram dataset of the six investigated models. Due to the low standard deviation, the confusion matrix in the first run almost entirely reflects the performance. The 2DS-CNN model achieved a mean precision and recall of 99.58% and 99.35%, respectively, while 2DM-CNN had a mean precision and recall of 99.78%. MLPC-CNN achieved 98.84% and 98.85%, respectively. AecNet is 98.83% and 98.85%. GoogLeNet and AlexNet have precision and recall of 99.69%, 99.68%, 98.83%, and 98.86%, respectively. These results show that the 2DM-CNN has the highest accuracy, demonstrating the effectiveness of the proposed solution, and AecNet has the lowest classification performance among the analyzed models.

**Fig 11:**
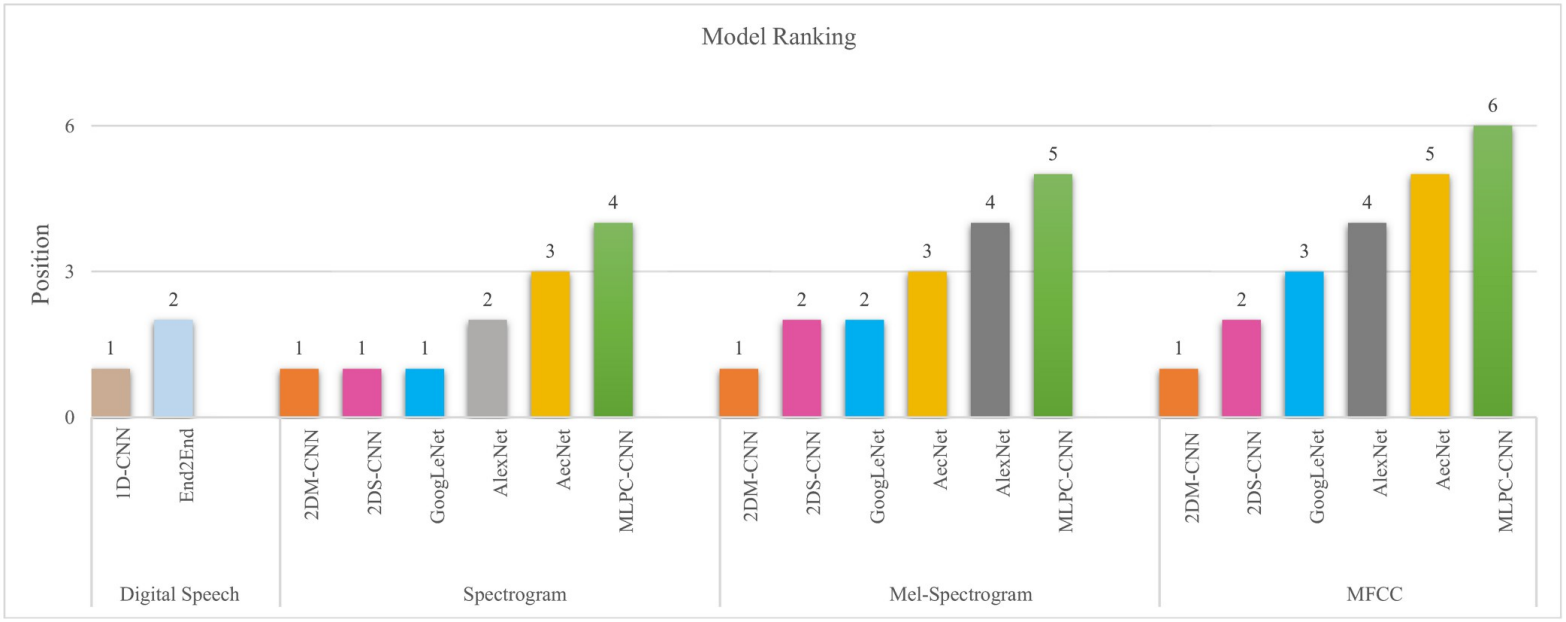
Models ranking (lower is better).

Fig 11 summarizes the results of all models on four different datasets. Based on the conformation of WRT, it can be concluded that the 2DM-CNN model has shown dominance over other models in the digital speech recognition problem. More detail, the 1D-CNN is better than the End2End model, and 2DM-CNN showed better performance than AlexNet, AecNet, and MLPC-CNN.

The 2DM-CNN model is tied with 2DS-CNN and GoogLeNet for the Spectrogram dataset. When looking at the *ρ*-value in the Wilcoxon statistic, we see that both 2DS-CNN and GoogLeNet have a *ρ*-value greater than 0.05 when compared to 2DM-CNN, indicating that the difference in accuracy is not significant enough to conclude that 2DM-CNN is the best model as shown in Table 6. For mel-spectrogram and mfcc datasets, the 2DM-CNN model has shown superiority over others. When working with complex representations like mel-spectrogram and mfcc, 2DM-CNN can extract features more effectively, thereby increasing the model’s accuracy.

## 6 Conclusions

In this paper, we have proposed digital speech recognition solutions based on three types of convolutional neural networks, including the 1D-CNN, 2DS-CNN, and 2DM-CNN. The obtained results show that the proposed 2DM-CNN model has the highest classification accuracy and performance on two types of input, mel-spectrogram, and mfcc, outperforms other models, equally ranking with the 2DS-CNN model on the spectrogram input type. The 1D-CNN model has lower classification accuracy than the two mentioned models but has the lowest learnable parameters and calculating time per testing set. Models using transfer learning techniques such as AecNet, GoogLeNet, and AlexNet have high classification accuracy but have a large number of parameters and computation times, making them unsuitable for low-performance devices. So, the proposed 2DM-CNN model can learn and classify digital speech better than others while saving computational resources and time.
